# The effect of glycopyrronium and indacaterol, as monotherapy and in combination, on the methacholine dose-response curve of mild asthmatics: a randomized three-way crossover study

**DOI:** 10.1186/s12931-017-0628-4

**Published:** 2017-08-02

**Authors:** Christianne M. Blais, Beth E. Davis, Donald W. Cockcroft

**Affiliations:** 1 Department of Physiology College of Medicine, University of Saskatchewan, 107 Wiggins Road, Saskatoon, SK S7N 5E5 Canada; 2 Department of Medicine, Division of Respirology, Critical Care and Sleep Medicine, University of Saskatchewan, College of Medicine, 103 Hospital Drive 5th Floor, Saskatoon, SK S7N 0W8 Canada

**Keywords:** Long-acting muscarinic antagonist, Ultra-long acting β_2_ agonist, Combination therapy

## Abstract

**Background:**

Methacholine dose-response curves illustrate pharmacologic bronchoprotection against methacholine-induced airway hyperresponsiveness and can be used to quantitate changes in airway sensitivity (position), reactivity (slope), and maximal responsiveness following drug administration. Our objective was to determine the influence of single-dose glycopyrronium (long-acting muscarinic antagonist) and indacaterol (ultra-long acting β_2_ agonist), as monotherapy and in combination, on the methacholine dose-response curve of mild asthmatics and to compare these findings with a non-asthmatic control curve.

**Methods:**

This was a randomized, double blind, double dummy, three-way crossover study. For asthmatic participants (*n* = 14), each treatment arm included a baseline methacholine challenge, drug administration, and repeat methacholine challenges at 1, 24, and 48 h. Non-asthmatic control participants (*n* = 15) underwent a single methacholine challenge and did not receive any study treatment. Methacholine dose-response curves were graphed as the percent fall in forced expiratory volume in 1 s (FEV_1_) for each methacholine concentration administered. Best-fit curves were then generated. Differences in airway reactivity were calculated through linear regression. Changes in airway sensitivity were assessed as the shift in the provocative concentration of methacholine causing a 20% fall in FEV_1_.

**Results:**

Compared to baseline, all treatments significantly reduced airway sensitivity to methacholine at 1 h post-dose (indacaterol ~1.5 doubling concentrations; glycopyrronium ~5 doubling concentrations; combination ~5 doubling concentrations). Bronchoprotection at 24 and 48 h remained significant with glycopyrronium and combination therapy only. Airway reactivity was not influenced by indacaterol whereas glycopyrronium significantly reduced airway reactivity at all time-points (*p* = 0.003-0.027). The combination significantly decreased slope at 1 (*p* = 0.021) and 24 (*p* = 0.039) hours only. The non-asthmatic control and 1-h glycopyrronium curves are nearly identical. Only the non-asthmatic control and 1-h post-combination therapy curves appeared to generate a true response plateau (three data points within 5%), which occurred at a 14% fall in FEV_1_.

**Conclusions:**

Methacholine dose-response curves differentiate the bronchoprotective mechanisms triggered by different classes of asthma medications. Assessment of bronchoprotection using methacholine dose-response curves may be useful during clinical development of respiratory medications when performing superiority, equivalence, or non-inferiority trials.

**Trial registration:**

clinicaltrials.gov (NCT02953041). Retrospectively registered on October 24th 2016.

**Electronic supplementary material:**

The online version of this article (doi:10.1186/s12931-017-0628-4) contains supplementary material, which is available to authorized users.

## Background

Research into the pathophysiology and treatment of asthma is complicated by this condition’s heterogeneity and its presentation as several phenotypes. Although treatments are thoroughly studied for their safety and efficacy through animal models and clinical trials, they often lack literature on physiological effects in human in vivo models. For example, the influence of new therapies on common diagnostic tests, such as methacholine challenge testing (MCT) for airway hyperresponsiveness, is often unknown.

The airway hyperresponsiveness component of asthma results from increases in both sensitivity and maximal responsiveness to bronchoconstrictors [1]. These effects can be visualized on a methacholine dose-response curve (MDRC); compared to healthy controls, the MDRC of asthmatics is shifted to the left (i.e. increased sensitivity to methacholine), has a steeper slope (i.e. increased airway reactivity), and either lacks or exhibits a raised plateau (i.e. excessive airway narrowing). Physiological factors that may increase airway sensitivity are epithelial damage or malfunction and increased inflammatory cell activity [1]. These factors may then contribute to abnormal autonomic cholinergic activity in the airways [1]. An increased maximal airway response to methacholine could in turn result from excessive smooth muscle contractility, increased levels of intraluminal secretions and/or airway inflammation [1].

The effect of respiratory medications can be elucidated through the changes they elicit on the MDRC, as this tool illustrates bronchoprotection produced against methacholine. For example, inhaled corticosteroids (ICS) have been found to produce a rightward shift and often a lower plateau [2–5]. Contrastingly, short- and long-acting β_2_ agonists (LABAs) only shift the MDRC to the right [6, 7]. Whether ultra-long acting β_2_ agonists (uLABAs) such as indacaterol produce the same result is unknown. Furthermore, a recent study found post hoc that a single dose of each of the long-acting muscarinic antagonists (LAMAs) tiotropium and glycopyrronium produced both a rightward shift and a significantly lower plateau on the MDRC [8].

Despite the fact that LAMA and uLABA medications are not used as monotherapy, the examination of their unique effects on the MDRC would reveal how they each contribute to bronchoprotection against methacholine. While no studies have examined the impact of LAMA/uLABA combination therapy on MCT results, such investigations could be beneficial for determining how they influence the test and whether any synergism is observed.

This study investigated the effects of glycopyrronium (LAMA) monotherapy, indacaterol (uLABA) monotherapy, and combination (combo) therapy on the MDRC of mild asthmatics. For comparison, a control group of non-hyperresponsive non-asthmatic adults was recruited to generate a “normal” MDRC. Methacholine dose shifts post-treatment were interpreted as a secondary measurement of drug-induced bronchoprotection against methacholine.

## Methods

### Participants

Participants were at least 18 years of age and provided written informed consent. This study was approved by the University of Saskatchewan Biomedical Research Ethics Board (Bio-REB 16-205) and was registered on clinicaltrials.gov (NCT02953041).

Eligible asthmatics had a provocative concentration (PC_20_) of methacholine causing a 20% fall in forced expiratory volume in 1 s (FEV_1_) ≤ 8 mg/mL, and baseline FEV_1_ (% predicted) ≥ 65%. ICS monotherapy was allowed if it had been taken regularly at a stable dose for 30 days minimum. Cholinergic agents and LABAs were avoided for 10 days, while salbutamol was avoided for 6 h prior to testing. Contraindications for the study treatments excluded individuals with a prostate, kidney or urinary retention problem, hypokalaemia, diabetes or glaucoma. Individuals were also ineligible if they had suffered allergen-induced asthma symptoms or an upper respiratory tract infection in the 4 weeks preceding the study, if they were pregnant or nursing, or if they had cardiovascular problems.

Eligible non-asthmatics required a negative methacholine PC_20_ (>16 mg/mL). A participant was excluded from analysis if they were found to be hyperresponsive (i.e. PC_20_ < 16 mg/mL).

### Methacholine challenge

MCT was performed with Provocholine® (Methapharm Inc., Brantford, ON, CA) according to the 2-min tidal breathing protocol [9]. Bennett Twin jet nebulisers (Puritan Bennett Corporation, Carlsbad, CA, USA) were calibrated to an output of 0.13 mL/min [9], and a participant used the same nebuliser for all of their testing. Post-treatment, MCT was stopped when the highest methacholine concentration (128 mg/mL) had been administered, when a response plateau had been achieved (providing FEV_1_ had fallen at least 10%), when a 40% fall in FEV_1_ occurred, or if the participant wished to stop. This study defined a plateau as three consecutive data points within 5% [4, 5, 10].

### Study design

This was a randomised, double blind, double dummy, three-way crossover study. A non-asthmatic study group served as a control for methacholine responsiveness.

Asthmatic participants completed three treatment arms, each consisting of three study visits at the same time of day (±2 h) on three consecutive days. On Day 1 of each treatment arm, a methacholine challenge was performed to establish a participant’s baseline methacholine response. Under observation, participants next self-administered one of the blinded treatments followed by MCT at 1, 24 and 48 h post-treatment. Each treatment was separated by at least 10 days. Non-asthmatic participants underwent a single methacholine challenge.

### Study drugs and blinding

Active treatment and placebo capsules were pre-loaded into Breezhaler® inhalers. Each treatment entailed administering the contents of two Breezhaler® devices. For the LAMA monotherapy, one device was loaded with a 50 μg glycopyrronium (Seebri®) capsule and the other a placebo capsule, for the uLABA monotherapy, one device contained a 75 μg indacaterol (Onbrez®) capsule and the other a placebo capsule, and for the combo therapy, one device contained a 50 μg glycopyrronium capsule and the other a 75 μg indacaterol capsule.

### Statistical analysis

Methacholine PC_20_’s were calculated by algebraic formula [11]. In the event that a 10-19.9% fall in FEV_1_ was achieved, the PC_20_ was extrapolated using the following formula: methacholine PC_20_ = [20/(last % fall in FEV_1_)] x last methacholine concentration (mg/mL) administered [12]. If a participant’s post-treatment FEV_1_ fell less than 10% after 128 mg/mL methacholine, the PC_20_ was arbitrarily set at 256 mg/mL.

A sample size of 15 asthmatics provides a study power of 99% for detecting a one-half concentration difference in methacholine PC_20_. Log-transformed PC_20_ data were used to assess the dose shift in methacholine responsiveness from baseline: dose shift = (Δlog_10_ methacholine PC_20_)/0.3 [13]. Clinically significant bronchoprotection was defined as a dose shift greater than one doubling concentration of methacholine [14].

MDRC slopes were determined through linear regression analysis of the data points at the two methacholine concentrations that had been administered prior to attaining the ≥20% fall in FEV_1_ at baseline, in addition to all remaining consecutive data points, or up to the first data point of a plateau [6]. Log-transformed slope values were then compared with paired t-tests.

A two-way (subject/treatment) analysis of variance and LSD all-pairwise comparisons were performed with Statistix 9 (Analytical Software, Tallahassee, FL, USA) to analyse differences in dose shift and baseline spirometry data (two-sided significance = 0.05) [15]. Graphs with best-fit curves were generated with SigmaPlot 10 (Systat Software, Inc., Richmond, CA, USA). If a response plateau developed, the maximal response was calculated by averaging the three plateau data points [16]. Results are presented with 95% confidence intervals unless otherwise stated.

## Results

### Participants

#### Asthmatics

Sixteen asthmatic participants enrolled in the study; fourteen completed all three treatment arms (Table 1). One participant was removed prior to treatment two due to significant improvement in their baseline PC_20_ > 16 mg/mL. One participant was removed prior to their third treatment due to poor asthma control. Reported side effects were mild and included tremors, cold-like symptoms, headaches, fatigue, dizziness, flushing, and throat irritation. These side effects are known to sometimes occur with the methacholine challenge or the study treatments, and all subsided without need for intervention. One participant took daily, stable-dose ICS therapy for the duration of the study.

**Table 1:** Asthmatic participants demographics

Participant	Gender	Age (years)	Height (cm)	BMI (kg/m^2^)	Mean FEV_1_/ FVC	Mean Baseline FEV_1_ (L)	Mean Baseline FEV_1_ (% Predicted)	Mean Baseline MCh PC_20_ (mg/mL)
01	F	24	157	21	0.79	2.24	74	0.24
02	M	40	178	27	0.75	3.49	83	0.92
03	M	20	169	22	0.71	3.61	84	3.6
04	F	28	168	23	0.69	3.32	97	3.8
05	M	27	173	29	0.61	3.14	73	0.22
06	F	23	173	22	0.81	3.55	95	2.0
07	F	21	163	20	0.91	3.70	111	2.4
08	F	21	173	30	0.86	3.65	98	3.7
09	F	29	160	20	0.86	2.62	84	2.0
10	M	32	185	32	0.67	3.41	72	1.8
11	M	70	168	23	0.67	2.14	78	1.7
12	M	22	173	27	0.78	4.17	95	3.1
13	F	18	130	21	0.87	1.62	73	1.3
14	M	25	172	27	0.82	4.23	99	2.1
15	F	30	160	25	0.78	2.98	96	3.2
16	M	23	180	24	0.89	4.29	90	2.4
Means:	50% Male	28 [12]	168 [12]	25 [4]	0.78 [0.09]	3.26 [0.77]	88 [12]	2.2^a^

*BMI* body mass index, *FEV*_*1*_ forced expiratory volume in 1 s, *FVC* forced vital capacity, *MCh* methacholine, *PC*_*20*_ provocative concentration of MCh causing a 20% fall in FEV_1_; [], standard deviation;^a^, geometric mean

Mean Baseline MCh PC_20_ – determined by averaging the pre-treatment baseline PC_20_’s

#### Non-asthmatics

Twenty-two non-asthmatic participants enrolled in the study. Fifteen met all eligibility criteria and were included in the analysis (Table 2). Of the seven excluded, six had a PC_20_ < 16 mg/mL and one had a significant airway response following saline inhalation.

**Table 2:** Non-hyperresponsive non-asthmatic participants demographics

Participant	Gender	Age (years)	Height (cm)	BMI (kg/m^2^)	Mean FEV_1_/FVC	Mean Baseline FEV_1_ (L)	Mean Baseline FEV_1_ (% Predicted)
H01	F	25	163	20	0.76	3.64	112
H02	F	22	168	21	0.83	3.10	88
H03	F	18	180	21	0.90	3.31	82
H04	F	36	163	20	0.78	2.57	83
H05	M	26	180	24	0.80	4.83	103
H06	M	20	170	30	0.78	3.33	77
H07	M	20	170	28	0.71	3.77	88
H08	M	27	170	33	0.85	4.78	115
H09	M	29	164	25	0.90	3.43	90
H10	M	34	175	30	0.84	4.15	98
H11	F	47	173	24	0.75	3.30	101
H12	F	20	170	30	0.81	4.00	111
H13	F	22	168	19	0.87	3.44	99
H14	F	35	157	26	0.81	3.30	113
H15	M	64	178	28	0.80	2.90	84
Means:	47% Male	30 [12]	170 [7]	25 [4]	0.81 [0.05]	3.59 [0.63]	96 [13]

*BMI* body mass index, *FEV*_*1*_, forced expiratory volume in 1 s, *FVC* forced vital capacity; [], standard deviation

### Methacholine dose-response curves

**Fig. 1:**
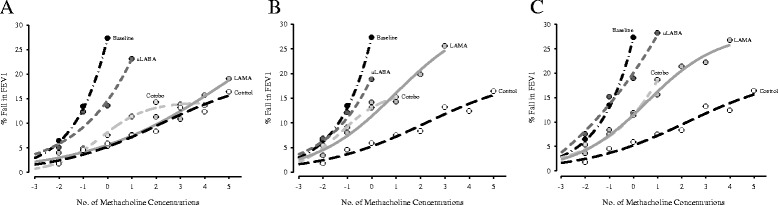
Mean non-asthmatic (control), baseline and post-treatment methacholine dose-response curves at 1 h (**a**), 24 h (**b**), and 48 h (**c**) are shown. The methacholine concentration causing a minimum 20% fall in FEV_1_ at baseline is designated as zero on the x-axis (i.e. corresponds to the final data point on baseline curves). Mean responses below (three) and above (five) comprise the post-treatment dose-response curves. Only data points with at least *n* = 8 are included. The sample size for each curve is: *n* = 15 for LAMA, *n* = 16 for uLABA, and *n* = 14 for combo

Mean asthmatic MDRCs for baseline, 1, 24, and 48 h post-treatment for each drug and the mean non-asthmatic control MDRC are depicted in Fig. 1 a-c. The mean non-asthmatic control MDRC and the mean combo MDRC at 1 h post-dose meet the study definition of a plateau (i.e. last three data points within 5%); both MDRCs formed a plateau at a 14% fall in FEV_1_.

In terms of mean slopes (m), the uLABA (m = 5.5, 6.0, and 6.6 at 1, 24, and 48 h, respectively) did not differ significantly from baseline (m = 10.4) at any time-point. Only the 1-h LAMA slope (m = 1.7) was statistically similar to the control slope (m = 2.0; *p* = 0.514). The LAMA at 1, 24 (m = 4.2), and 48 h (m = 3.8) differed significantly from baseline (*p* = 0.003, 0.027, and 0.016, respectively). The combo differed significantly from baseline at 1 (m = 3.0; *p* = 0.021) and 24 h (m = 3.3; *p* = 0.039), but not at 48 h (m = 4.4; *p* = 0.067). The 1-h post-dose individual MDRCs are illustrated in Additional file 1 and reflect the variability with respect to which treatment provided the more favourable response and how a specific treatment altered the characteristics of the MDRC in each participant. A short discussion on the supplementary figure can be found in Additional file 2.

### Bronchoprotection (Methacholine dose shift)

**Fig. 2:**
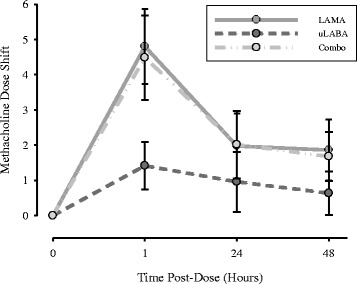
Mean methacholine dose shifts and their respective 95% confidence intervals for each treatment arm at 1, 24, and 48 h post-dosing. The sample size for each treatment is: *n* = 15 for LAMA, *n* = 16 for uLABA, and *n* = 14 for combo

Mean methacholine dose shifts from baseline at 1, 24, and 48 h post-treatment are illustrated in Fig. 2. Mean baseline PC_20_’s are 2.13 [1.32-2.95] for the LAMA, 2.47 [1.83-3.11] for the uLABA, and 1.78 [1.21-2.34] for the combo therapy. The uLABA and combo were statistically different (*p* = 0.024).

The LAMA and combo treatments provided clinically significant protection against methacholine-induced bronchoconstriction at 1, 24 and 48 h through dose shifts of approximately five, two, and two doubling concentrations, respectively. The uLABA only provided clinically significant bronchoprotection at 1 h with a dose shift of approximately 1.5 doubling concentrations. All dose shifts post-LAMA and post-combo are equivalent. Both treatments differ significantly from the uLABA at 1 (*p* < 0.00001) and 48 h (*p* = 0.012). The LAMA and uLABA differ significantly at 24 h (*p* = 0.049). Table 3 describes the proportion of participants receiving clinically significant bronchoprotection from each treatment over 48 h.

**Table 3:** Proportion of participants with a dose shift ≥1 doubling concentration post-treatment

Time Post-Dose	LAMA, *n* = 15	uLABA, *n* = 16	Combo, *n* = 14
1 h	100% (15/15)	62.5% (10/16)	100% (14/14)
24 h	73.3% (11/15)	37.5% (6/16)	64.3% (9/14)
48 h	80% (12/15)	18.8% (3/16)	64.3% (9/14)

LAMA, long-acting muscarinic antagonist; uLABA, ultra-long acting β_2_ agonist; Combo, combination therapy

### Bronchodilation

Baseline and post-treatment FEV_1_ data are described in Table 4. Mean baseline FEV_1_ results for the LAMA, uLABA and combo did not differ significantly (*p* = 0.060). No treatment produced significant bronchodilation; the combo produced the greatest improvement in FEV_1_ (160 mL increase from baseline at 48 h post-treatment).

**Table 4:** Mean baseline and post-dose shifts in FEV_1_ for each treatment arm

Treatment:	Baseline FEV_1_ (L)	FEV_1_ 1 h (L)	FEV_1_ 24 h (L)	FEV_1_ 48 h (L)
LAMA	3.25 [2.77-3.73]	3.31 [2.86-3.76]	3.33 [2.87-3.79]	3.26 [2.82-3.71]
uLABA	3.32 [2.91-3.72]	3.31 [2.91-3.72]	3.34 [2.94-3.73]	3.29 [2.89-3.69]
Combo	3.16 [2.70-3.62]	3.25 [2.77-3.72]	3.26 [2.78-3.74]	3.32 [2.83-3.81]

[], 95% confidence intervals; *FEV*_*1*_, forced expiratory volume in 1 s, *LAMA* long-acting muscarinic antagonist, *uLABA* ultra-long acting β_2_ agonist, *Combo* combination therapy

## Discussion

The investigation of drug effects through the changes they elicit in characteristics of the MDRC differentiates the three study treatments in terms of bronchoprotective mechanisms and overall derived benefits. With the glycopyrronium LAMA treatment, dose shift results demonstrate a large degree of bronchoprotection that lasted at least 48 h. These findings confirm those of a previous study and reinforce the fact that LAMAs could have clinical benefit in mild asthmatics [8]. FEV_1_ results were unsurprisingly small given that the study population likely has minimal airway constriction at rest. Interestingly, the LAMA MDRC at 1 h closely mimics the control MDRC, which suggests that of the three treatments, the LAMA-induced effects (i.e. largest rightward shift and reduction in slope) most closely resemble normal airway function. The significant decreases in airway sensitivity and reactivity as well as the mild reduction in maximal response were expected in part due to the direct antagonism of muscarinic receptors.

Several mechanisms beyond antagonism of muscarinic agonist binding may explain the MDRC changes observed. Decreased maximal airway responsiveness may result from inhibition of airway smooth muscle (ASM) contractility due to blockage of calcium release and signalling, which has been shown post-glycopyrronium in guinea pig trachea experiments [17]. LAMAs have also been found to influence airway remodelling, an important factor in airway hyperresponsiveness; the LAMA tiotropium prevents airway remodelling in guinea pig trachea preparations by inhibiting both airway wall thickening and hypertrophy of mucous glands [18]. However, airway remodelling may not be a significant factor within the context of this short single-dose study.

The significant LAMA-induced rightward shift could result from inhibition of abnormal autonomic cholinergic control (i.e. direct antagonism of methacholine and acetylcholine) and anti-inflammatory activity; in mouse models, glycopyrronium prevents the accumulation of inflammatory mediators such as interleukin-1β and tumour necrosis factor alpha following cigarette smoke exposure [19]. Tiotropium, which has been studied more extensively in animal models, inhibits the production of eosinophils and T-helper type 2 cytokines in mice acutely or chronically challenged with ovalbumin [20]. Nevertheless, whether inflammation is significant within the context of this study could be debated. Finally, the lower MDRC slope, which may be due to reductions in maximal responsiveness and airway sensitivity, reflects a significantly slower onset of airway symptoms during an exacerbation. Overall, the LAMA MDRCs suggest the importance of further studies into the bronchoprotective mechanisms triggered by this drug class in humans; such investigations could have important implications for their clinical use.

In contrast to glycopyrronium, the uLABA indacaterol produced a much lower degree and duration of effect. At 1 h, the uLABA improved methacholine tolerance by approximately one-third of that achieved with the other two treatments. In addition, most participants returned within one doubling concentration of their baseline PC_20_ by 24 h. The uLABA MDRCs also show little more than a mild rightward shift that is short-lived, and no significant improvement in slope. While indacaterol has not previously been examined in asthmatics for its degree and duration of bronchoprotection against methacholine, the uLABA olodaterol has been found to provide at least 32 h of significant bronchoprotection [21]. The population of asthmatics included in the current study may have been too mild to experience significant clinical benefit, or perhaps the dosage administered (the recommended dose for chronic obstructive pulmonary disease) was too low. FEV_1_ findings corroborate this theory, as β_2_ agonists are excellent bronchodilators and yet little improvement in FEV_1_ was recorded post-treatment in the study population. Significant ASM relaxation is expected with β_2_ agonist treatment, as β_2_ receptor activation stimulates the cyclic adenosine 3′,5′- monophosphate (cAMP)/protein kinase A (PKA) pathway and leads to inhibition of myosin-light-chain kinase [22, 23]. Despite this mechanism of action, our observations indicate that the physiological antagonism of airway narrowing triggered by indacaterol cannot outcompete methacholine-induced bronchoconstriction for long.

A possible secondary explanation for the rapid loss of uLABA monotherapy efficacy may be receptor desensitization; prolonged activation of β_2_ receptors as well as normal muscarinic type-3 (M_3_) receptor activity can trigger the desensitization of the β_2_ receptor-type. β_2_ agonists increase cAMP and PKA production and with time, the latter product begins to phosphorylate and uncouple β_2_ receptors from G_s_ proteins, preventing further receptor activity [24]. Meanwhile, M_3_ receptor stimulation by methacholine promotes G_q_ protein-mediated production of protein kinase C (PKC). PKC deactivates β_2_ receptors in the same fashion as PKA and reverses the inhibition of myosin-light-chain kinase [25].

Although no synergistic benefits with the combo therapy are perceived in the dose shift findings, the MDRCs show otherwise. While the LAMA and combo produce virtually identical dose shifts at each time-point, the combo produces a distinct MDRC at 1 h, as it forms a response plateau. This supports the expectation of synergism between the LAMA and uLABA. For instance, the inhibition of muscarinic receptors by the LAMA could significantly increase the function of the uLABA; by inhibiting muscarinic type-2 (M_2_) receptors, LAMAs disinhibit the blockage of cAMP formation and prevent the activation of G_i_ proteins that would otherwise oppose the β_2_-activated Gs proteins [26]. M_3_ receptor blockage also prevents PKC production, thereby reducing the desensitization of β_2_ receptors. The improvement in slope post-combo does not indicate synergism, as it is steeper than that observed post-LAMA.

A synergistic increase in the inhibition of ASM calcium signalling may explain the response plateau at 1 h post-combo. β_2_ receptor stimulation of PKA production leads to deactivation of inositol triphosphate (IP_3_) receptors, inhibiting calcium release from the sarcoplasmic reticulum (SR) [25]. PKA also promotes calcium/sodium exchange, which depletes intracellular calcium and stimulates both sodium-potassium ATPase and calcium-activated potassium channels [27–29]. The end-result is hyperpolarization of ASM cells and inhibition of airway contractility. LAMAs work similarly as their blockage of M_3_ receptors prevents IP_3_ production [30]. Without IP_3_, intracellular calcium levels do not increase sufficiently to trigger contractile activity [31]. Additionally, LAMA-induced inhibition of M_2_-stimulated G_i_ activity prevents cyclic ADP-ribose from mediating calcium release via ryanodine receptor channels in the SR [32]. Altogether, the uLABA and LAMA should together block airway contractility and consequently excessive airway narrowing to a greater degree than either monotherapy.

It is peculiar that the combo initially shows more airway responsiveness than the LAMA monotherapy at 1 h before developing a response plateau. Initial responsiveness may be the by-product of a uLABA-induced increase in airway cilia beat frequency. While the LAMA inhibits further mucus production, the activation of β_2_ receptors stimulates the cilia to move airway secretions up the trachea to be swallowed [33]. This could lead to temporary obstruction of airflow until secretions are cleared from the airways.

Synergism may also help to explain the greater improvement in FEV_1_ achieved post-combo. For example, the previously mentioned interactive effects between β_2_ and muscarinic receptors may increase β_2_ receptor activation, leading to increased ASM relaxation. However, it is also possible that the lower (albeit not significantly) mean baseline FEV_1_ pre-combo meant participants were slightly more constricted, allowing more room for improvement. Overall, an improvement in FEV_1_ of 160 mL is not clinically significant in this study population, as published guidelines for significant post-bronchodilator FEV_1_ improvement is a minimum of 200 mL [34].

This study possesses some limitations, predominantly due to its context. Investigations of drug effects after single dose may not accurately reflect what would be observed with long-term treatment. In addition, drug deposition may have been influenced by the delivery method; the combination glycopyrronium/indacaterol inhaler (Ultibro®) is only available with an indacaterol dosage of 110 μg while indacaterol monotherapy is only marketed in doses of 75 μg. To maintain the same drug dosages between treatment arms, the combination therapy was administered via two inhalers (one per monotherapy) instead of administering both compounds together through one device. Therefore, beyond differences in dose, drug deposition and study findings may differ depending on the method of administration of the combination therapy. The range of methacholine concentrations available and the safety rules used for determining when to stop a methacholine challenge also limit the extent to which plateau development can be examined on the MDRC. Finally, it should be noted that these MDRC results might not apply to other LAMAs, uLABAs, or LAMA/uLABA combinations. Nevertheless, important observations were made through the generation of MDRCs.

## Conclusions

Our findings provide important preliminary information on the usefulness of glycopyrronium and indacaterol alone and in combination in mild asthmatics based on their effects on the MDRC. While indacaterol produced little benefit, glycopyrronium and combination therapy both provided significant bronchoprotection with regards to reduced airway sensitivity and reactivity to cholinergic stimuli. Only combination therapy significantly protected against excessive airway narrowing through the formation of a response plateau. This physiological benefit is important for preventing asthma-related deaths. Future studies examining the physiological effects of the study drugs in human in vivo models would be beneficial for informing clinical decision-making, particularly given that asthma patients exhibit a wide range of phenotypes.

## Additional files

Additional file 1: Figure S1. A-P This figure illustrates 1-h post-dose individual asthmatic MDRCs and reflects the variability with respect to which treatment provided the more favourable response and how a specific treatment altered the characteristics of the MDRC in each participant. (PDF 283 kb)

Additional file 2: Supplementary discussion on the individual asthmatic MDRCs 1 h post-dose. This document contains the figure legend for Supplementary Figure 1 A-P (i.e. Additional file 1) and a short discussion on the figure and the conclusions that can be drawn from it. (DOCX 19 kb)

## Abbreviations

ASM: Airway smooth muscle
cAMP: Cyclic adenosine 3′, 5′ - monophosphate
Combo: Combination therapy
FEV_1_: Forced expiratory volume in 1 s
ICS: Inhaled corticosteroids
IP_3_: Inositol triphosphate
LABA: Long-acting β_2_ agonist
LAMA: Long-acting muscarinic antagonist
M_2_: Muscarinic type-2
M_3_: Muscarinic type-3
MCT: Methacholine challenge testing
MDRC: Methacholine dose-response curve
PC_20_: Provocative concentration of methacholine causing a 20% fall in FEV_1_
PKA: Protein kinase A
PKC: Protein kinase C
SR: Sarcoplasmic reticulum
uLABA: Ultra-long acting β_2_ agonist
