# Supplementary figures and images for: The effect of glycopyrronium and indacaterol, as monotherapy and in combination, on the methacholine dose-response curve of mild asthmatics: a randomized three-way crossover study
Source: Respir Res. 2017 Aug 2;18:146. doi: 10.1186/s12931-017-0628-4 (PMC5541419; doi:10.1186/s12931-017-0628-4)

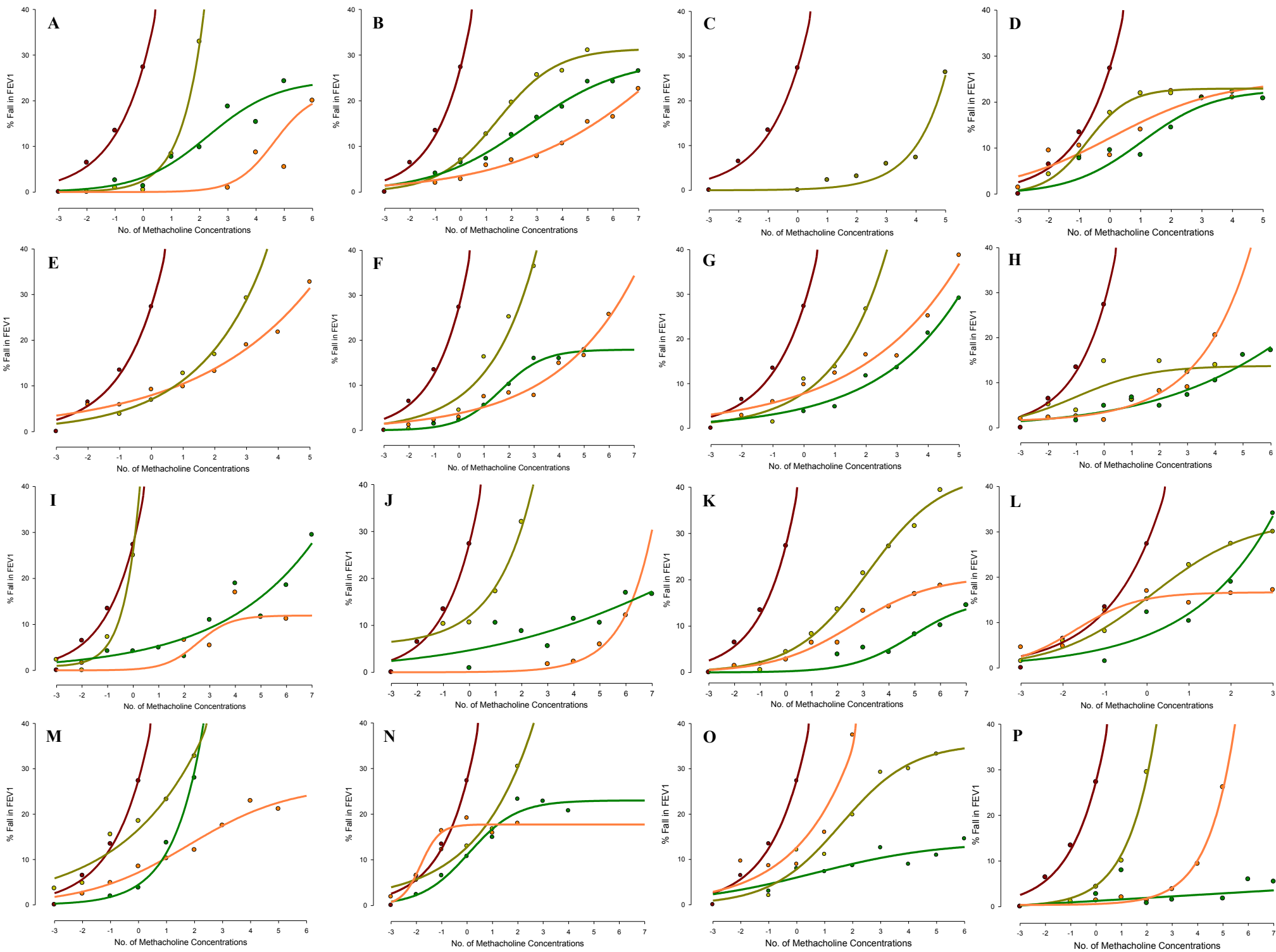

Supplement: Supplementary file 1 — A-P This figure illustrates 1-h post-dose individual asthmatic MDRCs and reflects the variability with respect to which treatment provided the more favourable response and how a specific treatment altered the characteristics of the MDRC in each participant. (PDF 283 kb) [file 12931_2017_628_MOESM1_ESM.pdf]
