# Supplementary material for: The effect of glycopyrronium and indacaterol, as monotherapy and in combination, on the methacholine dose-response curve of mild asthmatics: a randomized three-way crossover study
Source: Respir Res. 2017 Aug 2;18:146. doi: 10.1186/s12931-017-0628-4 (PMC5541419; doi:10.1186/s12931-017-0628-4)
Supplement: Supplementary file 2 — Supplementary discussion on the individual asthmatic MDRCs 1 h post-dose. This document contains the figure legend for Supplementary Figure 1 A-P (i.e. Additional file 1) and a short discussion on the figure and the conclusions that can be drawn from it. (DOCX 19 kb) [file 12931_2017_628_MOESM2_ESM.docx]

## Supplementary discussion on the individual asthmatic MDRCs 1 hour post-dose

## Figure Legend:

**Figure S1:** Comparison of the mean asthmatic baseline methacholine dose-response curve with the 1-hour post-treatment curves, graphed separately for each participant. The methacholine concentration causing a minimum 20% fall in FEV_1_ at baseline is designated as zero on the x-axis (i.e. corresponds to the final data point on baseline curves). Higher and lower methacholine concentrations administered are plotted as positive and negative x-values, respectively.

Red = mean baseline; yellow = uLABA; orange = LAMA; green = combo

## Supplementary Discussion:

Although drug development research typically focuses on mean data, it was noticed in this study that individual responses to the study medications varied significantly. Individual participants differed in terms of both the drug(s) producing the more favourable response and the changes in characteristics on the MDRC elicited by each treatment. For example, participant D experienced similar benefits with each treatment, while participant I responded very differently to each drug. Some participants did not reach a response plateau (i.e. three consecutive data points within 5%) with any treatment (i.e. A, G, J, M), while others developed a response plateau after one (i.e. B, F, H, I, K, L, O, P), two (i.e. N) or all three (i.e. D) treatments. The uLABA did not always appear to be the least beneficial medication (i.e. D, H, O). In the same fashion, the combo did not always appear to be the more beneficial treatment (i.e. A, B, I, J, M). These individual findings illustrate the relevance of modernizing health care to personalized medicine, as asthma treatments are not one-size-fits-all.

Asthma is a highly variable disease, as individuals experience differences in symptoms, triggers, pathophysiologies, environments, and genetic predispositions. As a result, it should not be surprising that patients respond more favourably to some medications over others. Future research should aim to identify a set of feasible procedures to perform in patients in order to more accurately determine which drug will be of most benefit for them. The ideal drug is particularly effective at minimizing excessive airway narrowing (i.e. reducing maximal responsiveness), as this symptom if unmitigated causes asthma-related deaths.
